# Determination of nutrient content, β-carotene, and antioxidant activity of *Moringa oleifera* extraction using organic solution

**DOI:** 10.5455/javar.2022.i590

**Published:** 2022-06-26

**Authors:** Ucop Haroen, Syafwan, Kiki Kurniawan, Agus Budiansyah

**Affiliations:** 1 Faculty of Animal Science, Jambi University, Jambi, Indonesia; 2 Reseach Center for Vaccine and Drugs Development, National Research and Innovation Agency, Jl. Raya Jakarta-Bogor Km 46, Cibinong, Indonesia

**Keywords:** Antioxidant, β-carotene, *Moringa oleifera*, organic solution

## Abstract

**Objective:**

The research was conducted to determine β-carotene and antioxidant activities and screening of phytochemical substances of *Moringa oleifera* extraction using organic solution.

**Materials and Methods:**

550 gm of M. oliefera leaf flour was macerated. This research was conducted by laboratory experiments using the maceration method. The extraction was performed using three kinds of solvents, which are n-hexane, ethyl acetate, and methanol; for 3 x 24 h, they were concentrated with a rotary evaporator. Then, the flavonoid, phenolic, β-carotene isolation, and antioxidant tests were conducted using the 2,2-diphenyl-1-picrylhydrazyl on each fraction (n-hexane, ethyl acetate, and methanol).

**Results:**

The results of weighing each concentrated extract from the maceration process of each fraction (n-hexane, ethyl acetate, and methanol) were 12.67, 35.67, and 49.29 gm, with the total phenolic content (1.4595 ± 0.361, 46.5489 ± 1.832, and 39.74574 ± 0.786) and total flavonoid content of each fraction (3.3056 ± 0.039, 58.6389 ± 2.051, and 48.9056 ± 0.0809), respectively. The antioxidant activity test on the crude extract from the ethyl acetate fractionation showed that the IC_50_ value was 30.309 mg/ml. The ethyl acetate fraction has a high total phenolic and flavonoid content. The results of the isolation of β-carotene from *M. oleifera* leaf flour were 0.4798 gm, or equivalent to 0.956% carotenoids.

**Conclusions:**

Based on the results of the research, *M. oleifera* leaves are identified to have a fairly high antioxidant activity, which is 30.309 mg/ml, resulting from the potential compounds in *M. oleifera* leaves that function as inhibitors of antioxidant activity, which are the groups of phenolic and flavonoid compounds.

## Introduction

Indonesia is a tropical country with high- and low-level plants rich in biodiversity. It is estimated that 17% of all species on the earth’s surface are found in Indonesia [1]. The Indonesian nation has utilized these rich natural resources as sources of food, food coloring, clothing, cosmetics, and medicines [2,3]. Indonesia has a reasonably high ecosystem diversity, and 47 types of natural ecosystems range from areas covered by ice sheets, lowlands, tropical forests, coral reefs, mangrove forests, and savanna ecosystems. In addition, 30,000 of the 250,000 tall plant species are found in Indonesia. Most of these tropical plants function as traditional medicinal and fodder ingredients, but the use of plants as fodder ingredients has not been explored optimally. One attractive plant in terms of phytochemicals, out of the other thousand species, is *Moringa oleifera*, which is one plant that quickly grows in tropical and subtropical regions, such as Indonesia, and is internationally well known as a nutritious plant.

The leaves of *M. oleifera* are in the shape of an oval to obovate with flat leaflets and small size, and they are compound leaves on one stem. Young *M. oleifera* leaves are usually light green and change to dark green as they age. Young *M. oleifera* leaves have a soft texture and the old ones have a slightly stiff and rigid texture. Young *M. oleifera* leaves have a slight bitter taste but are edible and nontoxic [4]. *M. oleifera* leaves are frequently used for research, both to determine the content of chemical compounds contained in them and to analyze their nutritional content [5,6]. *M. oleifera* leaves are rich in nutrients, including calcium, iron, protein, fat, vitamin A, and vitamins B and C [4,7]. In addition, *M. oleifera* leaves are also known to contain various amino acids, such as aspartic acid, glutamic acid, alanine, valine, leucine, isoleucine, histidine, lysine, arginine, phenylalanine, tryptophan, cysteine, and methionine [8]. Based on the phytochemical survey of *M. oleifera* leaves, it is known that the leaves contain secondary metabolites. The main secondary metabolite components contained in *M. oleifera* leaves are derivatives of phenolic compounds known to fight free radicals [9]. When the phenolic derivative compounds are taken out of the leaves of *M. oleifera*, the total amount is 1.6% [10].

In addition to it being proven that the main secondary metabolite components of *M. oleifera* leaves are derivatives of phenolic compounds that function to fight free radicals, a study of the nutritional value of *M. oleifera* leaves needs to be carried out because it is one of the most important factors that must be conducted to prove the nutritional value contained in *M. oleifera* leaves. Therefore, it is necessary to determine the nutritional value, especially the content of β-carotene, to test the antioxidant activity, and to screen for phytochemical compounds contained in *M. oleifera* leaves.

## Materials and Methods

### Ethical statement

Moringa leaf samples (M. oleifera) were taken from the *M. oleifera* plant growing in Jambi Province, and as much as 550 gm had been dried. Phytochemical profile testing of *M. oleifera* leaf samples was carried out at the Natural Organic Chemistry Laboratory of the Indonesia Institute of Science (LIPI), certified by the National Accreditation Committee, No: LP-767-IDN, Tahun 2017 (http://www.kan.or.id/index.php/2-other/180-direktori-klien-lab-penguji-12).

The tools used were distillation apparatus, rotary evaporator Heidolph WB 2000, UV-1700 series spectrophotometer, an electric water bath, oven, UV lamp at 254 nm and 365 nm, Kjedahl flask, Kjedahl tube, a set of distillation apparatus, biuret, TLC plate, aluminum foil, filter paper, and the glassware commonly used in laboratories.

The materials used were the samples of *M. oleifera* leaves, methanol (Merck), ethyl acetate (Merck), n-hexane (Merck), chloroform (Merck), iron (III) chloride (Merck), acetic anhydride (Fission), ammonia (Merck), concentrated sulfuric acid (Merck), acetic anhydride (Merck), crystalline iodine (Merck), and silica gel 60 F_254_ (Merck).

### Sample preparation

*Moringa oleifera* leaves were washed and air-dried for 5–10 days. Then, the dried *M. oleifera* leaves were blended and sifted to form a powder. The dried powder (550 gm) was macerated using solvents, n-hexane, ethyl acetate, and methanol in a row for 3 × 24 h; and was concentrated with a rotary evaporator to obtain the fractions of n-hexane, ethyl acetate, and methanol [11].

### Testing the total flavonoid, total phenolic, and DPPH content

The research method was a laboratory experiment using a sample of *M. oleifera* powder to test total flavonoids, total phenolics, and antioxidants using the 2,2-diphenyl-1-picrylhydrazyl (DPPH) method on each fraction (n-hexane, ethyl acetate, and methanol).

### Analysis of total phenolic

Total phenol analysis was determined using the Folin–Ciocalteu method [12]. The sample was extracted with 5 ml of methanol (85%), homogenized, and centrifuged at 3,000 rpm for 15 min to obtain the supernatant. The supernatant was separated from the filtrate by a filtering method to obtain a filtrate. Then, 400 µl of the filtrate was taken by pipette and put into a test tube, and then it was added to the Folin–Ciocalteu solution, which was vortexed until homogeneous. Next, it was let to stand for 6 min, and then 4.2 ml of Na2CO3 5% solution was added. After that, the sample was let to stand at room temperature for 90 min before measuring its absorbance using a UV-Vis spectrophotometer at a wavelength of 760 nm. Standard curves were made by dissolving gallic acid with various concentrations of 5–40 µg/ml. The total phenolic value was calculated using the following regression formula: *y* = *ax* + *b*.

### Analysis of total flavonoid

The sample was dissolved using methanol and iron powder (Fe), then HCl2 M was added so that it gave a red color to the solution, indicating a positive flavonoid. Then, the absorbance of the solution was measured using a UV-Vis spectrophotometer at a wavelength of 415 nm. We used a quercetin solution with a concentration variation of 0–40 µg/ml for the flavonoid comparison standards. The total flavonoid value was calculated using the following regression formula: *y* = *ax* + *b*.

### Antioxidant test analysis (DPPH)

The measurement of antioxidant activity of each extract against DPPH free radicals was based on the method of Yen and Chen [13]. The mixture of a solution of 0.5 ml of DPPH (1 mM in methanol) and the extract solution (10–200 μg in 2 ml of methanol) was shaken and stood at room temperature for 30 min to provide the optimum reaction time between DPPH and hydrogen atoms donated by the antioxidants. A wavelength of 515 nm was used to measure the resulting absorption. The difference in the amount of absorption between the blank and the sample was used to figure out the percentage of inhibition for the sample.

The damping effect of DPPH scavenging (%) = [1−(*A*_s_/*A*_0_)×100]

where *A*0 = blank absorbent and *A*s = sample absorbent. The percentage of DPPH attenuation activity was plotted against the sample concentration. The attenuation value of 50% (IC_50_) was calculated from the graph of the attenuation percentage to the sample concentration. The test was repeated twice; quercetin was used as a comparison. The reducing energy of *M. oleifera* leaf extract used for the test: 4 mg of the sample was weighed in a sample bottle, then dissolved in methanol 4 ml to obtain a mother liquor with a concentration of 1,000 µg/ml. Then, a dilution process was carried out as follows: concentration 200 μg/ml, 500 μl pipette of the sample solution; concentration 100 μg/ml, 250 μl pipette of the sample solution; concentration 50 µg/ml, 125 μl pipette of the sample solution; and concentration 10 μg/ml, 25 μl pipette of the sample solution. Each concentration was then filled up to 2.5 ml with methanol.

### Isolation and determination of β-carotene content

The isolation and determination of β-carotene content contained in the *M. oleifera* leaf samples were modified from standard carotenoid analysis procedures [14]. 50 gm of *M. oleifera* leaf powder samples were macerated using methanol for 6 h. Then, the extract was separated from the dregs. The methanol extract was then evaporated with the solvent and washed with 10 ml of saturated NaCl. This saturated solution of methanol and NaCl extract was partitioned with n-hexane solvent as many as 50 times, and a layer of methanol/NaCl separated it. The n-hexane filtrate was saponified with a 5% KOH/methanol mixture for 3 h. After that, the n-hexane layer (top layer) was separated with KOH/methanol precipitation. The n-hexane filtrate was partitioned with n-hexane solvent with the addition of a small amount of distilled water to wash the remaining KOH/methanol layer. Next, it was dried with anhydrous Na2SO4 and air-dried for 24 h. The β-carotene precipitation that forms is then weighed. The AOAC [15] procedure was used to perform a rough analysis of *M. oleifera* leaf flour to find out what nutrients it had.

## Results and Discussion

The results of the proximate analysis of the nutrient content of *M. oleifera* leaf flour can be seen in Table 1. The table shows that the nutrient content of *M. oleifera* leaf flour is quite high so that it can be used as an alternative fodder for livestock. In addition, *M. oleifera* leaves also contain secondary metabolites, such as phenolic and flavonoid compounds, that act as antioxidants [4]. A simple way to get secondary metabolites out of *M. oleifera* plants is to use the maceration method.

The maceration process of *M. oleifera* leaf powder with a total of 550 gm uses the extraction method. The maceration process starts with using nonpolar solvents to convert polar solvents. The finely chopped and air-dried samples were extracted by maceration using n-hexane (7 × 2 l), then were macerated with ethyl acetate (8 × 2 l), and finally with methanol (6 × 2 l) for 3 days consecutively. Each extract was concentrated using a rotary evaporator, so the n-hexane, ethyl acetate, and methanol fractions were obtained. Each concentrated extract from the maceration was collected and weighed with a total of 12.67 gm for the n-hexane fraction, which was greenish yellow in color. The ethyl acetate fraction, which was bright red in color, was weighed at 35.67 gm and the dark red methanol fraction was considered at 49.29 gm.

### Total phenolic content

The determination of total phenolic content was analyzed using the Folin–Ciocalteu method, which is measured at a wavelength of 770 nm. The phenolic standard used is gallic acid. The absorbance value of the gallic acid standard solution was made in the form of a calibration curve so that a linear regression equation could be obtained, which is as follows: y = 0.0477x –0.0848, with the correlation coefficient value (R2) of 0.9923. The total phenolic content was determined in three fractions, namely the methanol, ethyl acetate, and n-hexane fractions, with the total phenolic content shown in Table 2.

**Table 1.** Nutrient content of *Moringa oleifera* leaf flour.

Nutrient content	*Moringa oleifera* leaf powder
Dry matter (%)	88.93
Crude protein (%)	29.45
Crude fiber (%)	8.76
Crude fat (%)	8.41
Calcium (mg)	7.95
Energy (Kcal/100 gm)	307.30

Laboratory of Faculty of Animal Science, University of Jambi, 2020.

Based on the fraction that has the highest phenolic content, which is 46.5489 ± 1.832%, it can be concluded that for every 100 gm, the determination results of the total phenolic content of each fraction, the acetate fraction extract contains the phenolic equivalent of 46.5489 ± 1.832% gallic acid. Compared to the methanol and n-hexane fractions extract, the methanol fraction extract produced 39.74574 ± 0.786% phenolic compounds, while the n-hexane fraction extract produced 1.4595 ± 0.361% phenolic compounds. Thus, the ethyl acetate fraction is the best solvent to obtain phenolic compounds. Contrary to Kumbhare et al.’s [16] study, the content of phenolic compounds in *M. oleifera* stem bark extracted using methanol as a solvent produced the highest phenolic compounds, namely 50.72% w/w. Kefayati et al. [17] also reported that the methanolic extract of *Euphorbia splendida *Mobayen had the highest total phenolic compounds (TPC) values, 270.74 0.005 mg/gm. At the same time, the ethyl acetate fraction extract only produced a TPC of 208.54 ± 0.010 mg/gm [18], also reported the extraction of flower moon leaves (*Tithonia diversifolia*) on total phenolic compounds using ethanol as a solvent, producing 1.37% phenolic compounds.

On the contrary, ethyl acetate as a solvent had 1.28% phenolic compounds. Esmaeili et al. [19] reported that methanol extract showed the highest total phenolic content in Trifolium pratense L. (red clover) plants *in vivo* and *in vitro*, as well as callus tissue compared to ethyl acetate extract. This is because the complex formed by a moiety of phenolic compounds with carbohydrates and proteins can be more easily extracted in methanol than in other solvents.

### Total flavonoid content

Analysis of the determination of total flavonoid content using standard quercetin for each fraction can be used to determine the type of fraction that has antioxidant activity from *M. oleifera* leaf powder. The principle of the analytical method for determining the total flavonoid content is the reaction of the stable complex formation between aluminum chloride and the keto group on the C-4 atom and the hydroxy group on the C-3 or C-5 atom, which is adjacent to the flavone or flavonol group [14]. Total flavonoid content is determined using a spectrophotometer at a wavelength of 415 nm. The absorbance value of the quercetin standard solution was made in a calibration curve with a linear regression equation: *y* = 0.009*x* + 0.0124, with a correlation coefficient value (*R*^2^) of 0.9989. The determination of the total phenolic content of each of the *M. oleifera* leaf extracts is displayed in Table 3.

Based on the determination results of the total flavonoid content of each fraction, it is known that the ethyl acetate fraction extract has the highest flavonoid content, which is 58.6389 ± 2.051%. Compared to the methanol and n-hexane fraction extracts, the methanol fraction extract yielded 48.9056 ± 0.0809%, while the n-hexane fraction extract yielded 3.3056 ± 0.039%. Thus, the ethyl acetate fraction extract produced the most flavonoid compounds.

Contrary to Kefayati et al. [17], who reported that total methanolic extract showed the highest total flavonoid compounds values, which was 208.23 ± 0.007 mg/gm, while the ethyl acetate fraction yielded 65.80 ± 0.006 mg/gm, lower than that of the methanol fraction. Rahman et al. [18] reported that the extraction of flower moon leaves (T. diversifolia) using ethanol as a solvent produced 3.41 mg/gm, while ethyl acetate as a solvent produced flavonoid compounds of 2.21 mg/gm.

**Figure 1.**
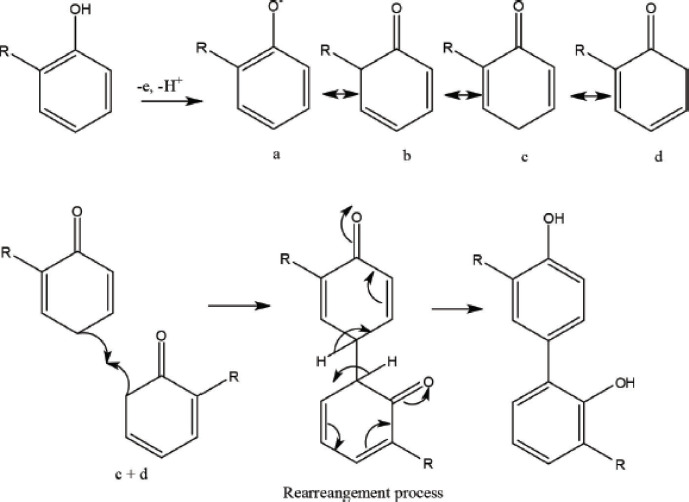
Formation, incorporation, and rearrangement reactions of phenoxyl radicals [40].

This study aims to determine the ability of *M. oleifera* leaf extract, which contains a group of phenolic and flavonoid compounds, to have an antioxidant activity that inhibits free radicals. It is affected by the hydrogen atoms in the hydroxy groups in the core of phenolic and flavonoid compounds, which act as hydrogen atom donors or free-radical fighters through an electron transfer process [20]. This causes the phenolic compounds to change into phenoxyl compounds (Fig. 1) through a rearrangement.

The preliminary tests conducted to determine the total phenolic and total flavonoid content contained in the *M. oleifera* leaf sample provide information that the crude extract from the ethyl acetate fraction has the highest total phenolic and total flavonoid values among the methanol and n-hexane fractions of 46.5489 ± 1.832 and 58.6389 ± 2.051, respectively. This demonstrates that the compound types of phenolic and flavonoid isolated in the ethyl acetate fraction are a group of semi-polar compounds. The study by Kefayati et al. [17] reported that the phenolic compounds have the strongest antioxidant activity in inhibiting free radicals. Moreover, Kumbhare et al. [16] said that antioxidant activity correlates with the content of total phenolic compounds. Methanolic, chloroform, and petroleum ether extracts at various concentrations ranging from 25 to 100 µg/ml were tested for their antioxidant activity using the DPPH radical scavenging assay method.

**Table 2.** Determination results of total phenolic content of each *Moringa oleifera *leaf fraction.

Fractions	Absorbance	Phenolic levels (%w/w)	Average + SD
n-hexane	0.0403	1.7148	1.4595 ± 0.361
	0.0283	1.2042	
Ethyl acetate	2.1269	45.2532	46.5489 ± 1.832
	2.2487	47.8446	
Methanol	1.8419	39.1894	39.74574 ± 0.786
	1.8942	40.3021	

Laboratory analysis of LIPI, 2021.

**Table 3.** Determination results of total flavonoid content of each *Moringa* *oleifera* leaf fraction.

Fractions	Absorbance	Flavonoid levels (% w/w)	Average + SD
n-hexane	0.0419	3.2778	3.3056 ± 0.039
	0.0424	3.3333	
Ethyl acetate	0.5532	60.0889	58.6389 ± 2.051
	0.5271	57.1889	
Methanol	0.4577	49.4778	48.9056 ± 0.0809
	0.4474	48.3333	

Laboratory analysis of LIPI, 2021.

### Antioxidant activity test of ethyl acetate fraction using DPPH method

**Figure 2.**
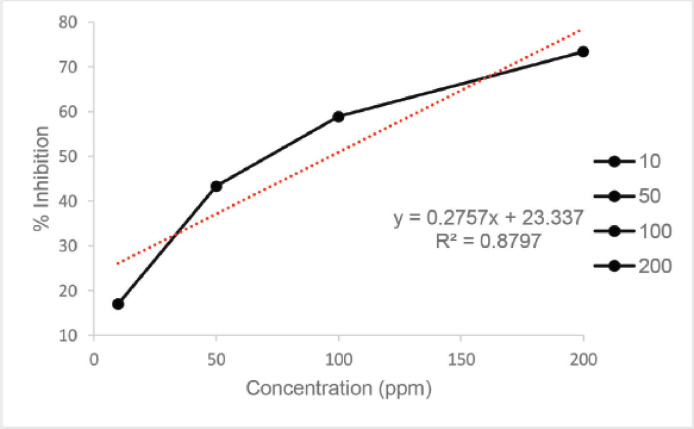
Graph showing the percentage of the inhibition versus concentration of ethyl acetate fraction extract.

**Figure 3.**
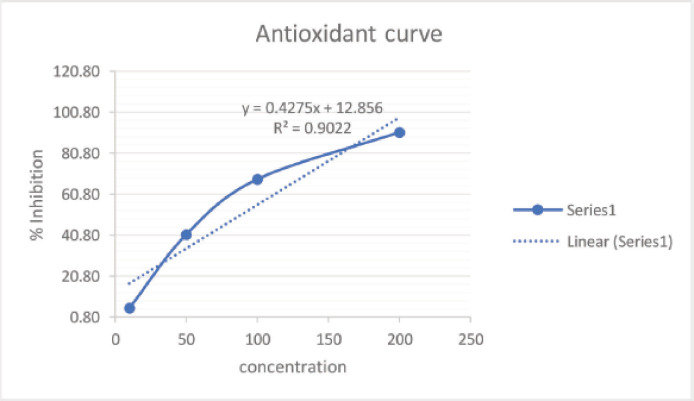
Graph showing the percentage of the inhibition versus concentration of methanol fraction extract.

**Figure 4.**
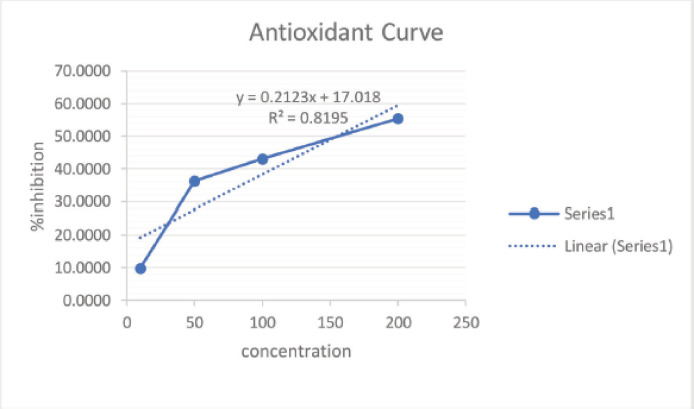
Graph showing the percentage of the inhibition versus concentration of n-hexane fraction extract.

Based on the information obtained, the determination of total phenolic and total flavonoid content has been conducted. The antioxidant activity testing focuses on the ethyl acetate fraction. The ethyl acetate fraction has a relatively high total phenolic and flavonoid content compared to the other fractions. The antioxidant activity testing was carried out using the DPPH method. The antioxidant activity testing on the crude extract, a fractionated result, is one of the primary test methods that provides information regarding the ability of natural compound extracts to inhibit free radicals (DPPH) by using a comparison compound with a quercetin-standardized solution. The determination of antioxidant activity is a quantitative test by capturing free radicals contained in DPPH compounds by the compounds that have a substituted-OH (hydroxy) group in the benzene core contained in natural material compounds, such as those in the group of phenolic and flavonoid compounds, using a UV-Vis spectrophotometer. The absorbance value resulting from the measurement of the UV-Vis spectrophotometer is converted into an IC_50_ value (inhibitory concentration), defined as the test compound concentration that can capture or stabilize free radicals as much as 50%. The smaller the IC_50_ value is, the more active the test compound will be against the antioxidant activity test. The graph showing the percentage of the inhibition versus. the concentration of ethyl acetate, methanol, and n-hexane fraction extracts can be seen in Figures 2, 3, and 4 and Table 4. Table 4.

**Table 4.** IC_50_ value of ethyl acetate fraction from *Moringa oleifera* leaf powder.

Concentration	Ethyl acetate fraction		Methanol fraction		N-hexane fraction	
	% Inhibition	IC_50_	% Inhibition	IC_50_	% Inhibition	IC_50_
10	17.1700	30.309	5.15	86.8865	9.8255	155.3556
50	43.4244		41.11		36.2393	
100	58.9808		68.08		43.0374	
200	73.2322		91.01		55.3945	

Based on the graph, it is known that the regression equation value is *y* = 0.2757*x* + 23.337, with a correlation coefficient (*R*^2^) value of 0.8797. The IC_50_ value obtained using the regression equation formula is 30.309 mg/ml. The IC_50_ value obtained from this experiment is relatively low, below 50 mg/ml, proving that the ethyl acetate fraction extract from *M. oleifera* leaf powder is strong enough to be used as an antioxidant activity [21]. The methanol fraction extract IC_50_ value was 80.8865 and the n-hexane fraction extract IC_50_ value was 155.3556. Atawodi et al. [22] reported that using the xanthine oxidase model system, extracts of roots, leaves, and stem bark of *M. oleifera* were extracted using methanol solvent and exhibited strong *in vitro* antioxidant activity with IC_50_ values of 16, 30, and 38 µl, respectively. Differences in the antioxidant content of *M. oleifera* were different due to differences in solvents due to varying types of antioxidants such as ascorbic acid, β-carotene, kaempferol, quercetin, rutin, isothiocyanates derived from leaves, tocopherol, myricetin, and lectins derived from seeds, palmitic acid, and phytosterols of flowers [23]. Kumbhare et al. [16] found that the bark extract of *M. oleifera* with a DPPH reagent of 78.49% produced the most antioxidant activity with a methanol solvent, followed by a chloroform fraction extract of 50.68% and a petroleum ether extract of 34.14%.

In poultry, antioxidant compounds help cope with stress [24–28], especially in countries with tropical climates, such as Indonesia and Southeast Asian countries, that consist of two seasons, namely dry and rainy seasons. In the dry season, high environmental temperatures cause poultry to experience stress (heat stress) [29,30]. To reduce this situation, poultry requires the intake of foods that contain antioxidants, such as vitamin C, and active compounds derived from plants or other natural ingredients [28,31,32]. Antioxidants in *M. oleifera* leaves are useful in reducing the stress conditions in this poultry, especially in broilers prone to stress due to extreme changes in environmental temperature. In goats, it was reported that *M. oleifera* leaves help increase ration consumption and the immune system through the transfer of bioactive compounds, especially antioxidants and vitamin C, in the milk [33]. In humans, *M. oleifera* leaf extract is pharmacologically helpful in medicine because it has anti-inflammatory, antioxidant, antitumor, anticancer, as well as antibacterial and antifungal activities [34]. The DPPH method is used to test the antioxidant activity of the ethyl acetate fraction. The IC_50_ value is found in Table 4.

### β-carotene content

Isolation of carotenoid compounds from *M. oleifera* leaf powder is important to determine the number of carotenoid compounds contained in *M. oleifera* leaf flour, which is as much as 0.4798 gm. Acknowledging the β-carotene content in the samples of *M. oleifera* leaf powder can function as additional nutrition and antibody enhancement for poultry as β-carotene is a source of pro-vitamin A [33–36]. β-carotene can also improve the reproductive performance of livestock, and the high content of antioxidant compounds will make livestock more resistant to disease [37–39]. In the form of an orange-red powder, the isolated carotene compound provides an observation. This study found that the β-carotene content in a 50-gm sample of *M. oleifera* leaf powder was 0.956%. Based on the calculation, the result is as follows: 


$$\%\;\mathrm{Carotenoids}=\frac{0.4798\;\mathrm{gm}}{50\;\mathrm{gm}}\times 100=0.956$$

## Conclusion

From the research, *M. oleifera* leaves are proven to have fairly high antioxidant activity, which is 30.309 mg/ml. This is because the *M. oleifera* leaves contain potential compounds that function as inhibitors of antioxidant activity, which are the groups of phenolic and flavonoid compounds. Phenolic and flavonoid compounds contained in the samples of *M. oleifera* leaf powder are determined by calculating the total phenolic content and total flavonoid content, which are 46.5489 ± 1.832 (%w/w) and 58.6389 ± 2.051 (%w/w), respectively.

## List of abbreviations

DPPH, 2,2-Diphenyl-1-picrylhydrazyl; IC, Inhibitory concentration; UV, Ultraviolet; nm, Nanometer; TLC, Thin-layer chromatography; rpm, Revolutions per minute; mM, Millimolar; AOAC, Official method of analysis; μl, Microliter; *R*^2^, Correlation coefficient value; %w/w, Percentage of weight per weight.
